# Efficacy of Internet-Based Cognitive Behavioral Therapy for Depression and Anxiety among Global Population during the COVID-19 Pandemic: A Systematic Review and Meta-Analysis of a Randomized Controlled Trial Study

**DOI:** 10.3390/healthcare10071224

**Published:** 2022-06-30

**Authors:** Maria Komariah, Shakira Amirah, Emir Gibraltar Faisal, Stephanie Amabella Prayogo, Sidik Maulana, Hesti Platini, Suryani Suryani, Iyus Yosep, Hidayat Arifin

**Affiliations:** 1 Department of Fundamental Nursing, Faculty of Nursing, Universitas Padjadjaran, Sumedang 45363, Indonesia; 2 Faculty of Medicine, Universitas Indonesia, Jakarta 40115, Indonesia; shakira.amirah@ui.ac.id (S.A.); emir.gibraltar@ui.ac.id (E.G.F.); stephanie.amabella@ui.ac.id (S.A.P.); 3 Faculty of Nursing, Universitas Padjadjaran, Sumedang 45363, Indonesia; sidik17001@mail.unpad.ac.id; 4 Department of Medical-Surgical Nursing, Faculty of Medicine, Universitas Padjadjaran, Sumedang 45363, Indonesia; hesti13001@unpad.ac.id (H.P.); hidayat.arifin@unpad.ac.id (H.A.); 5 Department of Mental Health Nursing, Faculty of Nursing, Universitas Padjadjaran, Sumedang 45363, Indonesia; suryani@unpad.ac.id (S.S.); iyusyosep0607@gmail.com (I.Y.)

**Keywords:** anxiety, COVID-19, depression, internet cognitive behavioral therapy

## Abstract

**Background:** Depression and anxiety have become the most common mental health disorders worldwide during the COVID-19 pandemic, and increasing interest in telemedicine has led to the innovation of using internet-based cognitive behavioral therapy (iCBT). **Objective:** This systematic review and meta-analysis aimed to evaluate the efficacy of iCBT for depression and anxiety among the global population during the COVID-19 pandemic. **Methods:** A literature search was conducted on PubMed, Scopus, Cochrane, ProQuest, Wiley, and Web of Science using the PRISMA framework, and only randomized controlled trials (RCTs) were included in the study. A critical appraisal was also performed using Cochrane’s Risk of Bias (RoB) 2. The meta-analysis used random-effects models to analyze pooled mean difference (MD) and its *p*-value. **Results:** Twelve RCTs were included for qualitative analysis and nine RCTs, which yielded 6778 patients with depression and 6556 patients with anxiety during the COVID-19 pandemic, were included for quantitative analysis. Despite high heterogeneity, all studies had a low risk of bias. Pre- and post-iCBT intervention in the depression forest plot depicts a significant effect (*p* < 0.00001) with a pooled MD of 4.73 (95% CI: 4.55–4.90), while the pre- and post-iCBT intervention depicts a significant effect (*p* < 0.00001) with a pooled MD of 4.50 (95% CI: 4.34–4.67). This demonstrates that iCBT was found to significantly decrease depression and anxiety scores in patients during the COVID-19 pandemic. However, substantial heterogeneity was also found (I^2^ = 93%; *p* < 0.00001 and I^2^ = 90%) for the pre-/post-depression and anxiety forest plots, respectively. **Conclusions:** This meta-analysis comprises an evidence-based result for iCBT to treat depression and anxiety in the COVID-19 population, as indicated by the significantly lower assessment scores. Delivering iCBT in this situation needs to be considered more extensively, as it has promising results and yields the benefits of technological advancement in psychotherapy.

## 1. Introduction

Mental health has become one of the most important health indicators that leads to one in five people living with a disability. The World Health Organization (WHO, 2017) reports that mental health disorders are increasing worldwide, with an increase of 13% in the last decade [1]. Depression and anxiety have become the most common mental health disorders worldwide, accounting for 3.4% and 3.8%, respectively [2]. Santomauro et al. [3] calculated an additional 532 million cases of major depressive disorders worldwide (a 276% rise) due to the COVID-19 pandemic, resulting in a cumulative prevalence of 31,529 instances per 100,000 people. They also calculated 762 million different instances of anxiety disorders globally (a 256% rise), resulting in a prevalence of 48,024 cases per 100,000 people [3]. In a meta-analysis of the prevalence of depression during the COVID-19 outbreak, Bueno-Notivol et al. [4] revealed that it was seven times greater than in 2017, which highlights the great impact of the pandemic on people’s psychological problems (2021).

People tend to suffer from sadness, stress, and anxiety during a health crisis, due to the panic of being infected with the disease [5,6,7,8]. People who suffer from diseases that do not have a cure or immunization will experience worry, tension, depression, and anxiety [8]. As a result of the COVID-19 pandemic, people have experienced psychological discomfort and mental health concerns [8], and early action should be taken to maintain health during a pandemic, especially for people who suffer from anxiety and depression.

Depression is a mood disorder characterized by a depressed mood and loss of interest or pleasure in all or almost all activities, which can impair day-to-day functioning [9]. Anxiety is a mental disorder that produces unpleasant worries or concerns about future events or fear of reacting to current events, and also interferes with daily functioning [10]. Depression and anxiety are associated with suffering, disability, poor health, and high societal costs [11] and are correlated to other comorbid illnesses such as cardiovascular disease, obesity, and diabetes [12,13,14]. Depression and anxiety can cause sleep disturbance, which has a substantial connection with mortality and morbidity over time and can negatively impact people’s health and general quality of life [15,16,17]. Therefore, treating these illnesses is critical for optimizing the management of patients’ quality of life, and scalable mental health supports and interventions are urgently needed.

Pharmacological and psychological treatments are generally used for mental health disorders. Medications, such as serotonin reuptake inhibitors (SSRIs) and tricyclic antidepressants (TCAs), continue to be a mainstay in treating depression and anxiety, but patients’ adherence rates are low, possibly due to their side effects [10,18,19]. Psychological therapy is preferred by patients over medications, [20] and psychotherapy, such as cognitive-behavioral therapy (CBT), is implemented to maintain and manage psychological problems. CBT is used because it is the only psychotherapy intervention with evidence of efficacy and is recommended for depression and anxiety [18,19,21].

The COVID-19 pandemic disrupted traditional face-to-face mental health treatments, including CBT, resulting in the widespread implementation of services via telephone and videoconferencing [22,23,24,25,26]. For example, internet-based cognitive behavioral therapy (iCBT) was an effective addition to traditional mental health services [22], and the program used the same principles as CBT via electronic learning facilities and structured assignments. iCBT has high accessibility and confidentiality, where users can access learning and feedback anytime and anywhere [27]. iCBT has various delivery techniques, accompanied by different features that support the success of programs that take place in the short term with specific objectives. The program can be self-guided and/or guided by health professionals. The program’s content and delivery also vary according to the characteristics of the patient [28]. According to some studies, there was an increase in demand for digital mental health services throughout the pandemic; therefore, using iCBT for anxiety and depression has to be explored due to the predicted rise in anxiety and depression caused by COVID-19. Consequently, we conducted this systematic review and meta-analysis to evaluate the efficacy of iCBT for depression and anxiety among the global population during the COVID-19 pandemic.

## 2. Materials and Methods

### 2.1. Study Design

This systematic review and the meta-analysis were conducted according to the Cochrane Handbook for Systematic Reviews of Intervention, following the Preferred Reporting Item for Systematic Review and Meta-analysis (PRISMA) framework (Appendix A) [29]. We used this study design to provide a more precise estimate of effect size and increase the reliability (precision) of the estimated iCBT intervention [30]. This study was registered in the PROSPERO (CRD42022310734).

### 2.2. Search Strategy

**Figure 1:**
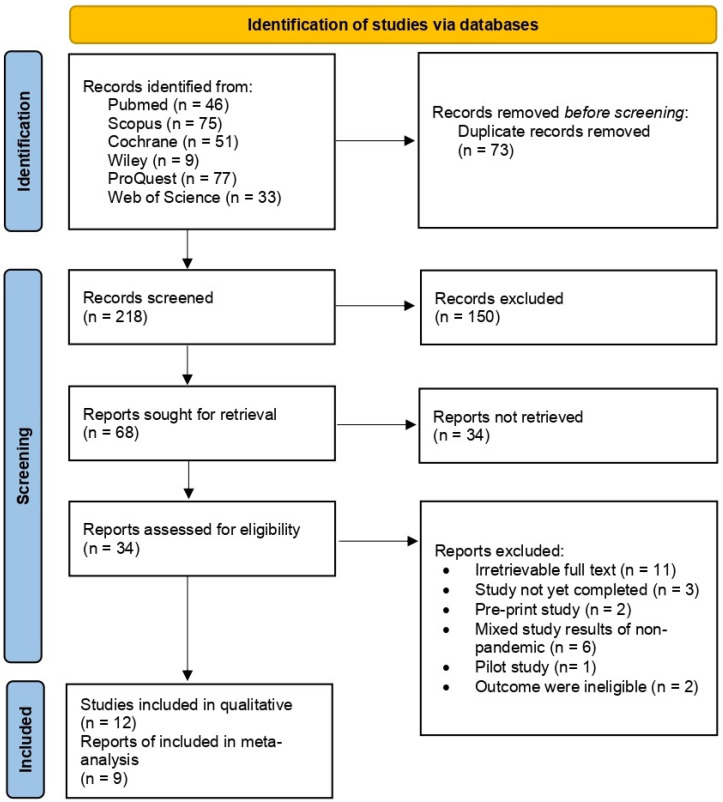
PRISMA flow diagram.

We conducted a comprehensive literature search on PubMed, Scopus, Web of Science, Cochrane, Wiley Library, and ProQuest using the PRISMA framework. We collected data on 25 January 2022, using the keywords “Internet cognitive behavioral therapy” OR “iCBT” AND “Depression” OR “Anxiety” AND “COVID-19” OR “Pandemic”. All the terms matched the medical subject headings (MeSH) Browser. Figure 1 presents the PRISMA flow diagram.

### 2.3. Eligibility Criteria

The inclusion criteria followed the PICO framework (patient/problem, intervention/exposure, comparison/control, outcome) and comprised (1) type of study: RCTs; (2) study population: patients with depression and anxiety during the COVID-19 pandemic; (3) intervention: iCBT; (4) outcomes: depression and anxiety scores using respective tools in mean, standard deviation, and *p*-value for pre- and post-intervention and control; and (5) pre-treatment or other care as control. Meanwhile, the exclusion criteria were set to (1) studies that were not complete at the time of retrieval; (2) studies with irretrievable full-text articles; and (3) studies in languages other than English as an international language. Furthermore, duplicate removal was also performed using EndNote X9 software. The titles and abstracts of studies were screened according to criteria of accessibility by three independent reviewers (SA, EGF, and SAP). Any disagreements were discussed to consensus.

### 2.4. Data Extraction

We extracted the included studies using a predetermined outcome sheet in tabular form, which consisted of (1) author and year of publication; (2) study characteristics, including study location, study period, and study design; (3) study population, including sample size, mean/range age, and psychosocial condition; (4) intervention, including the name of the intervention, frequency, the assessment tool used, and duration to follow-up; and (5) study outcomes, including the efficacy of iCBT on depression and anxiety during the COVID-19 pandemic in terms of its mean difference and significance (*p*) values, and patients’ adherence. Study characteristics were assessed qualitatively by two reviewers (SA and EGF), and another author (SAP) rechecked the accuracy of the extracted data while performing statistical analysis.

### 2.5. Quantitative Data Analysis

Statistical analysis was performed using Review Manager ver. 5.4 (The Nordic Cochrane Center, The Cochrane Collaboration, Copenhagen, Denmark). Mean differences and standard deviation with a 95% confidence interval (CI) and *p*-value were extracted from studies for both pre- and post-intervention and intervention versus control post-treatment. We then interpreted pooled effects using random-effects models. The main results used in the statistical analysis were the mean difference between pre- and post-treatment using iCBT for patients with depression and anxiety during the COVID-19 pandemic, which was shown by lower depression and anxiety scores in the respective tools used, as well as the mean difference between iCBT and control group patients. The mean difference with a 95% CI and its respective *p*-value was used to determine the efficacy of iCBT on depression and anxiety patients during the COVID-19 pandemic, which is presented in a forest plot. We used an inverse variance and DerSimonian–Laird random-effects model as proposed by Riley et al. [31], as we considered that heterogeneity outside the study could also be discovered. Heterogeneity was further evaluated using I2 statistics based on the Cochrane threshold, with cut-off limits of 0%, 25%, 50%, and 75% as insignificant, low, moderate, and high heterogeneity, respectively [32]. We also performed sensitivity analysis following Duval and Tweedie’s trim-and-fill method to identify any outlier study if high heterogeneity was detected.

## 3. Results

### 3.1. Study Selection

We found 12 eligible studies for systematic review and extracted 9 for meta-analysis; 10 studies were extracted for depression meta-analysis and eight for anxiety meta-analysis [22,33,34,35,36,37,38,39,40,41,42,43]. These studies were included because they contained sufficient data for quantitative analysis. The included studies are described in Figure 1 [29].

### 3.2. Characteristics of Included Studies

Twelve RCTs yielding 6778 patients with depression and 6556 patients with anxiety during the COVID-19 pandemic, who were treated with iCBT, were included for quantitative and qualitative analysis. The studies were conducted in several locations (Australia, China, Germany, Sweden, Norway, Israel, Italy, Oman, and South Africa) and published during the COVID-19 pandemic from 2020 to 2021. Additionally, all studies’ outcomes were obtained from June 2020 to December 2020 and followed up over six weeks. The mean age of participants was 22, and they were further randomized into the intervention group and control group, or several follow-up intervention groups. Interventions involved iCBT and were conducted over several sessions, which were then assessed with standardized tools, including the Patient Health Questionnaire-9 (PHQ-9), Beck Depression Inventory-II (BDI-II), Hamilton Rating Scale for Depression (HAMD-17), Generalized Anxiety Disorder (GAD-7) questionnaire, Short Health Anxiety Inventory (SHAI), State Anxiety Inventory-Form Y1 (STAI-Y1), and Hamilton Anxiety Rating Scale (HAMA) questionnaire [22,33,34,35,36,37,38,39,40,41,42,43]. Detailed literature search procedures are presented in Table 1.

### 3.3. Study Outcome

To analyze the studies, we conducted a meta-analysis on the PHQ-9 for patients with depressive disorders and GAD-7 for patients with anxiety disorders to achieve similar outcomes.

#### 3.3.1. Outcome of Pre- and Post-iCBT Intervention Efficacy for Patients with Depression during the COVID-19 Pandemic

**Figure 2:**
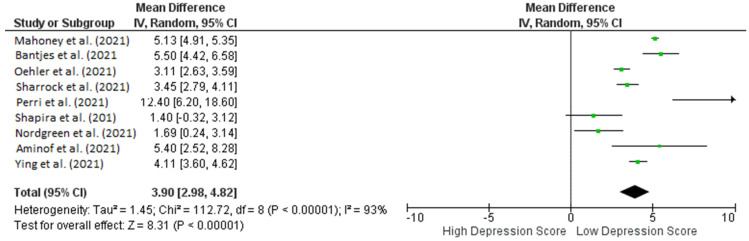
Forest plot pre- and post-intervention of ICBT in achieving low depression scores in depression patients during COVID-19 pandemic.

A summary of the study’s outcome is outlined in Table 2. The parameter for assessing efficacy was a low depression score in the questionnaires of the respective standardized tools. A meta-analysis assessed the pre- and post-intervention efficacy of iCBT, and the results are shown in a forest plot in Figure 2, which depicts a significant effect (*p* < 0.00001) with a pooled MD of 3.74 (95% CI: 2.83–4.65). This shows that iCBT was found to significantly decrease depression scores in patients with depression during COVID-19. Heterogeneity was also found (I^2^ = 93%; *p* < 0.00001).

#### 3.3.2. Outcome of Pre- and Post-iCBT Intervention Efficacy for Patients with Anxiety during the COVID-19 Pandemic

**Figure 3:**
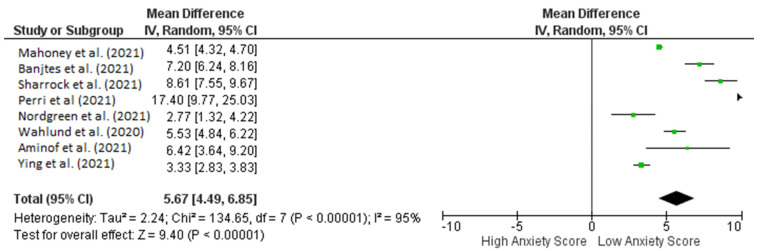
Forest plot pre- and post-Intervention of ICBT in achieving low anxiety scores in anxiety patients during COVID-19 pandemic.

Patients with anxiety who were treated with iCBT had lower anxiety scores in terms of pre- and post-intervention efficacy when GAD-7 was used as a screening tool. The pre- and post-iCBT intervention forest plot in Figure 3 depicts a significant effect (*p* < 0.00001) with a pooled MD of 4.84 (95% CI: 3.85–5.83), which shows that iCBT was also found to significantly decrease anxiety scores in patients during the COVID-19 pandemic. Heterogeneity was also found (I^2^ = 93%; *p* < 0.00001).

#### 3.3.3. Subgroup Analysis of Depression Scores Based on Clinically Guided or Self-Guided Intervention

**Figure 4:**
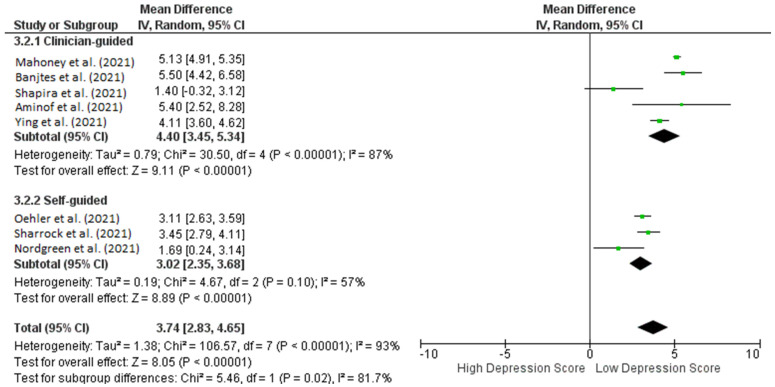
Subgroup analysis for studies analyzing pre- and post-ICBT intervention in depression based on methods of intervention (clinician-guided vs. self-guided).

Subgroup analysis shows that studies with clinically guided iCBT had superior outcomes compared to self-guided iCBT in terms of pre- and post-intervention. This resulted in the same low depression scores (Figure 4), indicating a promising and novel way to conduct interventions with patients diagnosed with depression during the pandemic (pooled MD: 4.40 [3.45, 5.34] versus 3.02 [2.35, 3.68]). The between-group difference is significant (*p* < 0.00001).

#### 3.3.4. Subgroup Analysis of Anxiety Score Based on Clinically Guided or Self-Guided Intervention

**Figure 5:**
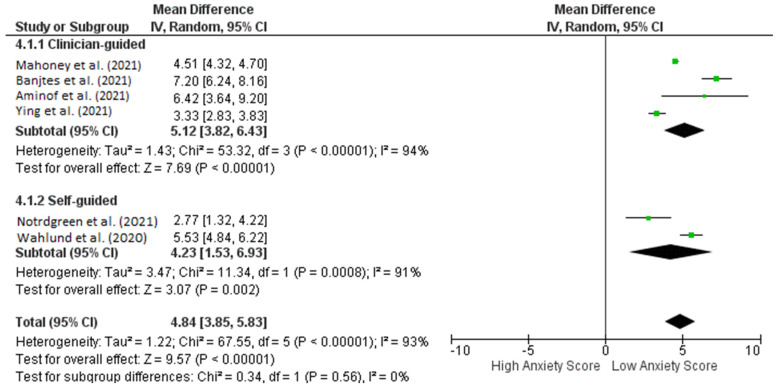
Subgroup analysis for studies analyzing pre- and post-ICBT intervention in anxiety based on methods of intervention (clinician-guided vs. self-guided).

Subgroup analysis shows interesting results for anxiety scores and indicates that self-guided iCBT had a superior outcome compared to clinically guided iCBT in terms of pre- and post-intervention, resulting in the same low anxiety scores (Figure 5). This highlights a promising and novel way to conduct interventions with patients diagnosed with anxiety during the COVID-19 pandemic (pooled MD: 5.12 [3.82, 6.43] versus 4.23 [1.53, 6.93]). The between-group difference is significant (*p* < 0.00001).

### 3.4. Sensitivity Analysis

We performed a sensitivity analysis, which, as explained in Cochrane’s book, is a repeat of the primary analysis or meta-analysis, substituting alternative decisions or value ranges for decisions that were arbitrary or unclear.

**Figure 6:**
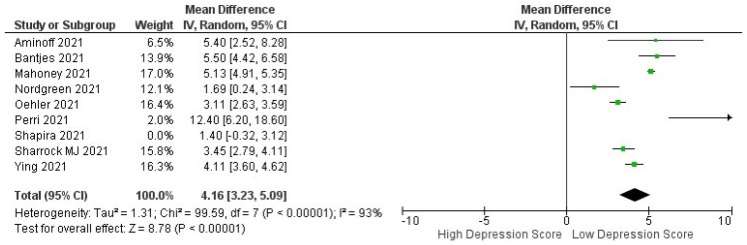
Sensitivity analysis for studies analyzing pre- and post- in depression score.

**Figure 7:**
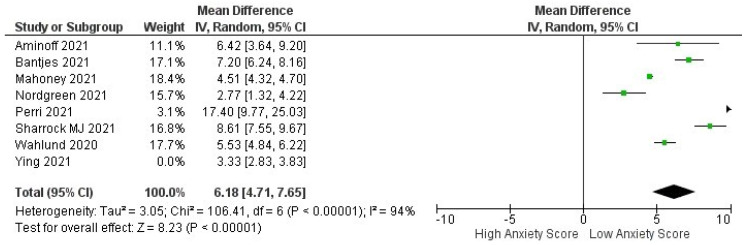
Sensitivity analysis for studies analyzing pre- and post- in anxiety score.

Based on the forest plot mentioned above, the included studies showed substantial heterogeneity. Thus, sensitivity analysis using Duval and Tweedie’s trim-and-fill method revealed that one study was an outlier. When Stav et al.’s pre- and post-analysis depression score was removed, the outcome of the trim-and-fill sensitivity analysis was an MD of 4.00 [3.08, 4.91], *p* < 0.00001; I^2^ = 94% (Figure 6). Moreover, pre- and post-analysis of the anxiety score was included in the sensitivity analysis, and the study by Yuchen et al. was found to be the outlier. When this study was removed, the outcome of the trim-and-fill sensitivity analysis was an MD of 5.22 [4.02, 6.42], *p* < 0.00001; I^2^ = 91% (Figure 7).

### 3.5. Publication Bias

**Figure 8:**
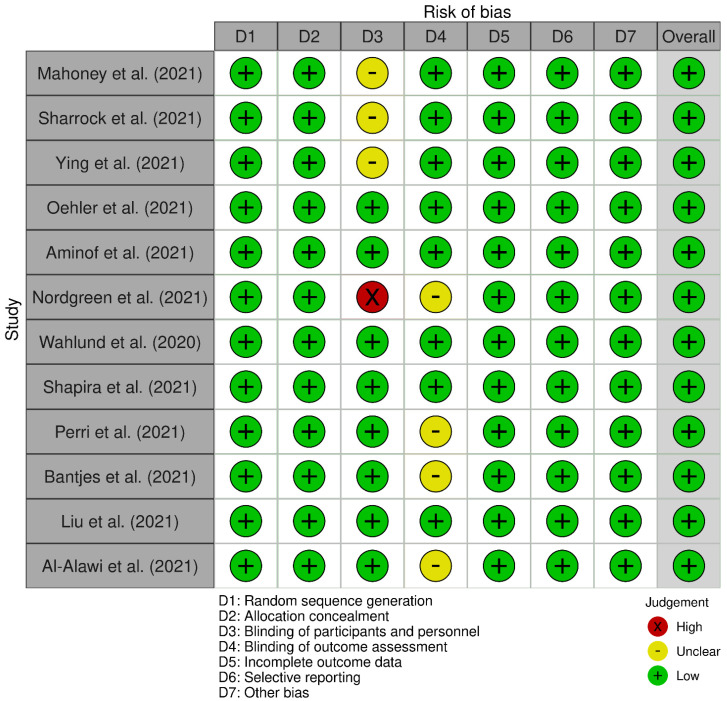
Traffic light plot’s risk of bias.

**Figure 9:**
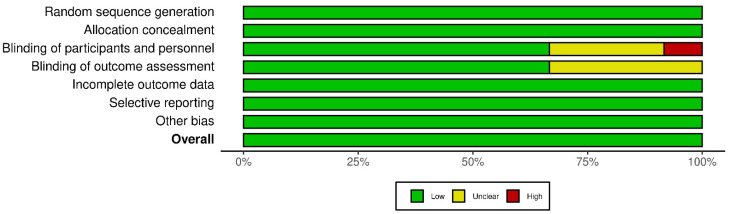
Summary risk of bias.

**Figure 10:**
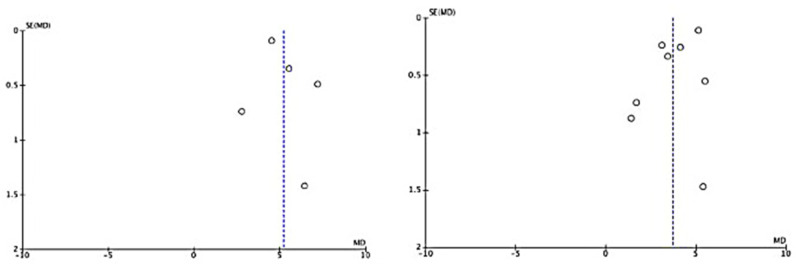
Funnel plot presenting publication bias and heterogeneity analyzes of pre- and post-intervention of ICBT for depression patients (**left**) and anxiety patients (**right**) during COVID-19 pandemic calculated in meta-analysis. The small circle represent individual study.

Each study was quality assessed using Cochrane’s RoB 2. Five of the included studies were of good overall quality, but Tine et al., Bantjes et al. [43], Sharrock et al. [33], Perri et al. [42], Ying et al. [36], and Mahoney et al. [22] showed considerable bias. This caused concern in domains that included no or unclear blinding of participants and personnel and the blinding of outcome assessment. Overall, the included studies were mostly of good quality (Figure 8 and Figure 9). Additionally, funnel plot analysis revealed a symmetrical plot, suggesting a lower bias of publication and homogeneity of studies (Figure 10).

## 4. Discussion

### 4.1. Principal Results

Telehealth, as a potential solution for this situation, has been supported by various studies, and with the increase in depression and anxiety during COVID-19, we would certainly benefit from increasing the use of and access to telehealth. First, telehealth is accessible to individuals in rural areas that have limited mental health resources. iCBT provides a way of removing one of the barriers to rural communities, as the patient-to-primary care physician ratio is still small (39.8:100,000). Furthermore, it lessens the distance traveled to reach the nearest health facility [44] as 20% of people who need mental healthcare do not have access to services. Second, telehealth demonstrates its versatility among clinical conditions that can lessen mental healthcare disparities. Third, various results have shown that telemedicine is more effective and efficient at delivering therapy as it is less time-consuming and there is less distance between patients and healthcare providers, making it more convenient and patients more compliant when accessing treatment [45,46]. By pooling the data obtained from RCTs during the COVID-19 pandemic, we analyzed the effectiveness of iCBT in reducing anxiety and depression and revealed lower assessment scores respective to each measurement tool.

Interestingly, our meta-analysis had an equal number of treatment outcomes as the interventions’ sub-analysis in the type of guidance, which was clinician-guided and self-guided (Figure 3 and Figure 4). The meta-analysis comprised the same screening tools within various studies to measure symptom severity for depression (PHQ-9) and different types of anxiety (GAD-7) [47,48]. For the screening tools’ effectiveness, a prospective non-controlled cohort study by Titov et al. [49] used the same assessment instruments and demonstrated that iCBT was an effective treatment for anxiety and depression with outcomes comparable to the results of controlled clinical trials of iCBT and with benchmarks of face-to-face CBT.

CBT is a physiological treatment for populations that are affected by mental health issues, including depression, anxiety, mental problems, etc. Several studies have stated that the advantages of CBT include improvements in functioning and quality of life. The CBT concept involves mastering our thoughts, feelings, and physical sensations, and it encourages people to become aware that their feelings are developed by their interpretation of the situation rather than the situation itself [50]. CBT is also included in the treatment strategy for depression in combination with pharmacotherapy [51,52].

In contrast to conventional CBT, which has boundaries and requires face-to-face interaction with patients, iCBT uses the internet and electronic devices, which allows patients to access interactive websites or software that provides them with relevant and friendly media. Whether conducted synchronously or asynchronously, patients with depression and anxiety have gained cost benefits compared to conventional methods. The major key to this intervention is its remote approach, as a self-help approach can help patients learn how to treat themselves in case of recurrence. In this meta-analysis, our data comprise consistent results in line with the current meta-analysis [52]. Theoretically, traditional CBT should improve certain comorbid conditions in anxiety and depression. Andersson et al. compared the effectiveness of guided online and face-to-face CBT among patients with somatic psychiatric disorders and concluded that the face-to-face therapist is not as crucial as stated in various literature [53,54]. Moreover, within the included studies, iCBT had more propitious results with high quality, which implies the feasibility of iCBT to overcome challenges in the near future. An RCT conducted by Hedman et al. [55] suggests that using iCBT with patients diagnosed with an anxiety disorder as a complementary therapy, not a substitute, for conventional CBT will facilitate the patient’s needs.

Interestingly, the iCBT results for lower depression and anxiety comorbidity scores are consistent. We meta-analyzed Yuan et al.’s RCT, which evaluated the efficacy of iCBT with stable effect sizes using a between-group or within-group comparison for depression and anxiety, and found that iCBT had a positive effect on both comorbid disorders [56]. A meta-analysis of Etzelmueller et al.’s study also supports the acceptability and effectiveness of guided iCBT for the treatment of depression and anxiety in a routine healthcare system [57]. The study elucidated the large effect of iCBT with the average pre-post effect size (Hedges’ g) of all anxiety interventions, including interventions that targeted both anxiety and depression, which was g = 0.94 (95% CI 0.83–1.06). However, in this everlasting pandemic, no meta-analysis has produced consistent results when evaluating the extent of the effect of iCBT among the population with depression and anxiety. This breakthrough research produces consistent results when using iCBT, with substantial heterogeneity for depression (80%) and anxiety (94%), which implies that the random effects of the meta-analyses weight studies nearly equally, regardless of sample sizes, and yields a meta-analytic summary close to the more easily calculated arithmetic mean of individual study results.

An important aspect of evaluating iCBT intervention requires adherence to be taken into consideration. Adherence rates for guided iCBT for depression and anxiety are significant for measuring the acceptability of the intervention and are related to the treatment outcome in trials. In this study, adherence can be described as the extent to which individuals are exposed to the content of the intervention. These findings are formed by dividing the mean number of completed sessions or modules by the maximum number of sessions or modules that apply to every evaluation of either CBT or iCBT intervention [58]. Interestingly, our meta-analysis evaluated adherence, and the results are provided in Table 2. The results show that adherence serves as a good predictor and moderator, which suggests that clinician-guided delivery is more acceptable than self-guided intervention [22,33,36,37,38,39,40,41,42,43]. This type of information can be beneficial in considering the implementation strategy.

Another interesting element that must be discussed is self-guided versus guided iCBT. We performed a subgroup analysis that separated the results of iCBT with and without guidance from health professionals. Results from the meta-analysis showed a pooled MD of 5.12 [3.82, 6.43] versus 4.23 [1.53, 6.93], and the between-group difference is significant (*p* < 0.00001). Unguided iCBT has the advantage of being more scalable and affordable, but previous studies by Cuijpers et al. (2019) [59] have shown that guidance generally results in better outcomes. To date, no studies have discussed which populations benefit from guided or unguided iCBT.

The strengths of this meta-analysis are its exclusive focus on evaluating iCBT interventions for their acceptability and clinical outcomes in real-world conditions. Unlike the previous meta-analyses that mix efficacy with effectiveness trials, in this review, we only focused on studies conducted in regular care settings during the pandemic [60]. The authors believe that, as the mental health situation escalates, the results of this meta-analysis will be important as we strive to report routine care results free from bias and possibly introduced within efficacy studies, such as the stricter application of protocolized procedures, eligibility criteria, and randomization. Another advantage of this meta-analysis is that there are no dropout rates in the 10 studies, which is to the advantage of iCBT, as it covers the vast majority of the depression and anxiety population. Nevertheless, the findings of this study should be interpreted with caution due to several limitations. The authors also presented an overview of iCBT implementation to increase its feasibility and gain a better understanding of how iCBT can be adopted by regular care services.

### 4.2. Limitations

Although iCBT is efficient for reducing symptoms, it requires further study. The RCTs included in this study were of high quality and the results were relatively stable, but heterogeneity may have influenced the final results, and potentially confounding factors may have affected their reliability. The sample sizes of several included studies were small, and iCBT therapy varied in the number and duration of therapy sessions, which may have yielded different efficacies in improving anxiety and depression symptoms. Furthermore, rating scales with different sensitivities were applied to assess anxiety and depression, which may have resulted in inconsistent outcomes. For methodological reasons, several limitations exist in this meta-analysis. First, the potential studies searched were published in English. Second, unpublished studies were not included, and although publishing bias was not observed in this meta-analysis, some unpublished studies might be important. In summary, we identified the effect of iCBT on comorbid anxiety and depression, despite heterogeneity and the limitations of the meta-analysis. Additional high-quality studies with larger sample sizes are needed to provide further evidence.

Third, the high level of heterogeneity poses a significant challenge to this review’s findings; therefore, we addressed heterogeneity to ensure that the data taken from the trial reports were correct and had a random effect. Finally, the population of this study was exceedingly diverse with too many different groups of people, which made it difficult to generalize the results to the specific population of interest.

### 4.3. Implications for Clinical Practice

Increasing demand for the treatment of common mental health disorders, such as depression and anxiety, presents a major public health challenge. Wittchen et al. estimated that nearly 40% of the population will require treatment for a mental health burden, namely, anxiety and depression, at some time during their life [61]. iCBT is an effective psychological therapy for stress and depression, and to obtain the best outcomes, physicians and nurses must understand that iCBT can frequently be used in combination with pharmacological therapy. Patients suffering from psychiatric problems must be referred to a mental health nurse who can counsel them on treatment choices. Primary care physicians are advised to collaborate with behavior therapists to implement and track the progress of iCBT.

However, problems can exist with ineffective treatments in psychotherapy and psychopharmacology [61], and in seeking diagnosis and access to treatment, many difficult challenges and structural barriers contribute to the failure of physiological treatment [62]. With adequate background knowledge and reasoning, choosing iCBT should be considered a solution. The most recent studies of the efficacy of iCBT as routine care can alleviate some of the challenges in implementation, including the need to improve and address the informal integration of healthcare systems to resolve the perceived skepticism toward iCBT from general practitioners. Second, the stable recruitment of patients as referral models to uphold effective communication strategies and improve work programs to attract patients to iCBT. Third, therapists’ working conditions need to incorporate developmental models of training, standards, and peer feedback during supervision [63]. Finally, long-term sustainability is the most important aspect concerning the transition to serving at the national level [57].

Of course, in some cases, iCBT will have the opposite effect, and relationships with therapeutic patients may be affected. Additionally, using the internet reduces face-to-face interactions, which may be helpful for patients with comorbid, demotivating, or other changes in circumstances. However, this can still be minimized by increasing the interaction between doctors and patients to achieve quality service that focuses on patient recovery. Other considerations are iCBT algorithms and the determination of the number of modules and session durations that provide maximum benefit for the patient [64].

## 5. Conclusions

In conclusion, both guided and unguided iCBT have been shown to reduce depression and anxiety scores during the pandemic. Taking no action to address the burden of major depressive and anxiety disorders should not be an option [3]. Based on the main problems and the evidence in our current global state, one innovative approach to resolve the problem is utilizing telehealth. Despite its limitations, this meta-analysis produces the evidence-based result that using iCBT during the COVID-19 pandemic reduces depression and anxiety scores and discusses the feasibility of its implementation in a healthcare setting. Our references did not find additional symptoms while using iCBT; therefore, it is implied that iCBT is safe and beneficial. The current approach to delivering iCBT content needs to be considered further. As an innovation, iCBT has potential, and its promising results yield the benefits of technological advancement in psychotherapy.

To enhance the quality of trials, follow-ups must be initiated, patients’ feedback must be collected, and studies could take place in developed and under-developed countries with established guidelines or assessments. Furthermore, it is necessary to increase the awareness of depression and anxiety, which is also often a problem during a pandemic. The public must be more aware of how people may experience life during the COVID-19 pandemic and the effect it has on their mental health. By increasing awareness, people can understand that depression and anxiety need to be treated as early as possible, and iCBT can be an affordable alternative solution for everyone. In the future, a holistic approach is needed regarding depression, anxiety, and iCBT, starting with increasing awareness, educating patients and families, and providing information to increase the use of iCBT as an alternative solution.

From this study, we recommend a further systematic review with meta-regression analysis to examine the moderation of iCBT. Furthermore, long-term results with a follow-up of more than six months are required to investigate the effectiveness of iCBT for depression.

## Figures and Tables

**Table 1:** Characteristic of included studies.

Authors, Year,	Study Location	Study Design	Population			Intervention				Quality
			Sample Size	Mean/Range Age (Years)	Psychosocial Condition	Intervention	Frequency	Assessment	Follow Up	
Mahoney et al. (2021) [22]	Australia	RCT	5074 Female (3557) Male (1311) Unspecified (332)	37.31 (13.53)	Adults experiencing symptoms of anxiety and depression	THIS WAY UP (thiswayup.org.au) iCBT	self-guided or guided by the end-user’s clinician, users granted 90 days access to complete their course	Depression: PHQ 9 Anxiety: GAD-7	-	6/7 ***
Sharrock et al. (2021) [33]	Australia	RCT	778 Female (526) Male (220) Unspecified (332)	37.76 (12.64)	Adults experiencing symptoms of anxiety and depression	THIS WAY UP (thiswayup.org.au) iCBT	Ninety days. There is a five-day wait-period between lessons two to six to give participants time to practice the skills covered in the lessons.	Depression: PHQ-9 Anxiety: SHAI	-	6/7 ***
Ying et al. (2021) [36]	China	RCT	127 Female (87) Male (40)	73.39 (7.37)	Symptoms of depression, anxiety, general psychological distress, and functional disability	Healthy Psychological Station (iCBT clinician guided)	Post-treatment (5 weeks)	Depression: PHQ-9 Anxiety: GAD-7	1 month	6/7 ***
Oehler et al. (2021) [37]	Germany	RCT	1423 Female (946) Male (477)	40.15 (13.35)	guided sample of patients with depression	iFightDepression tool (iFD), a web-based CBT intervention	at least two workshops in the first six weeks	Depression: PHQ-9	-	7/7 ***
Aminof et al. (2021) [38]	Swedia	RCT	26 Female (19) Male (7)	42.1 (16.8)	Participant with elevated levels of psychological distress	7-week-long individually tailored ICBT	Individual with 13 times completion module for 7 weeks	Depression: PHQ-9 Anxiety: GAD-7	-	7/7 ***
Nordgreen et al. (2021) [39]	Norway	RCT	82 Female (65) Male (17)	40 (14.19)	Self-reported depressive and anxiety symptoms and change in positive and negative emotions.	Self-guided Internet-delivered intervention integrated with The person-based approach (PBA; Yardley et al., 2015)	a new module every third day (3- day group, a total of 28 days) or a new module every fifth day (5-day group, a total of 40 days).	Depression: PHQ-9 Anxiety: GAD-7	6 weeks	5/7 ***
Wahlund et al. (2020) [40]	Swedia	RCT	335 Female (277) Male (58)	45 (13)	Daily uncontrollable worry about COVID-19 and its possible consequences (e.g., illness, death, the economy, one’s family)	3 weeks, Self-guided online cognitive behavioral intervention	Self-guided module, tasks to practice during at least a couple of days for 3 weeks	Anxiety: GAD-7	4 weeks	7/7 ***
Shapira et al. (2021) [41]	Israel	RCT	82 Female (66) Male (16)	72 (5.6)	community-dwelling older adults	iCBT via Zoom	online sessions via the Zoom video conferencing platform were delivered to groups of 5–7 people during seven sessions over 3.5 weeks each lasting between 1–1.5 h	Depression: PHQ-9	1 month	7/7 ***
Perri et al. (2021) [42]	Italy	RCT	19 Female (13) Male (6)	52.4 (10.6)	subjects requiring psychological support to manage the ongoing trauma associated with quarantine, isolation or work in COVID-19 hospital wards	trauma-focused cognitive-behavioral therapy (TF-CBT) using skype	received a 7-session therapy for a total duration of about 3 weeks (2 sessions per week)	Depression: BDI-II Anxiety: STAI-Y1	1 month	6/7 ***
Bantjes et al. (2021) [43]	South Africa	RCT	158 Female (135) Male (23)	22.4 (4.9)	Web-based groups were being offered to help students learn psychological skills to reduce symptoms of anxiety and depression	Web-based (Microsoft Teams) group cognitive behavioral therapy	10 weekly workshops of 60–75 min.	Depression: PHQ 9 Anxiety: GAD-7	4 weeks	6/7 ***
Liu et al. (2021) [34]	China	RCT	326 Female (102) Male(150)	IG: 43.76 (14.31) CG: 41.52 (11.51)	COVID-19 patients had mild to moderate depression or anxiety symptoms.	Computerized cognitive behavioral therapy (cCBT)	10 min per day to self-directed individual therapy for 1 week	Depression: HAMDA-17 Anxiety: HAMA	4 weeks	7/7 ***
Al-Alawi et al. (2021) [35]	Oman	RCT	46 Female (36) Male (10)	28.51 (8.70)	People in the community with anxiety and depression	iCBT	6 sessions of therapist-guided online or self-help therapy for week 6	Depression: PHQ-9 Anxiety: GAD-7	6 weeks	6/7 ***

**Note.** ^(^***^)^ Low; BDI = Back’s Depression Inventory; GAD = General Anxiety Disorder; iCBT = Internet-Based Cognitive Behavioral Therapy; HAMA = Hamilton Anxiety Scale; HAMDA = Hamilton Depression.

**Table 2:** Study outcome.

Study	Sub Study	Efficacy							Adherence
		Pre			Post			*p*-Value	
		Mean	SD	Total Patients	Mean	SD	Total Patients		
Mahoney et al. (2021) [22]	Depression	14.11	6.13	5074	8.98	5.07	5074	<0.001	Clinician guided: 30.8% Self-guided: 20.1%
	Anxiety	11.79	5.20	5074	7.28	4.36	5074	<0.001	
Sharrock et al. (2021) [33]	Depression	9.54	7.50	778	06.09	5.74	778	<0.001	Clinician guided: 31.43% Self-guided: 30.08%
	Anxiety	29.15	11.13	778	20.54	10.18	778	<0.001	
Ying et al. (2021) [36]	Depression	10.57	1.42	127	6.46	2.57	127	<0.001	Clinician guided: 87.4%
	Anxiety	9.51	1.31	127	6.18	2.55	127	<0.001	
Oehler et al. (2021) [37]	Depression	13.83	4.85	940	10.72	5.66	940	<0.001	Self-guided: 83,1%
Aminof et al. (2021) [38]	Depression	11.23	5.19	26	5.83	5.42	26	0.076	Clinician guided: 61.6%
	Anxiety	11.46	5.56	26	5.04	4.61	26	0.01	
Nordgreen et al. (2021) [39]	Depression	9.23	04.07	82	7.54	5.34	82	<0.05	Self-guided: 61%
	Anxiety	9.40	4.17	82	6.63	5.25	82	<0.001	
Wahlund et al. (2020) [40]	Anxiety	13.93	4.10	335	8.40	4.95	335	<0.001	Self-guided: 60%
Shapira et al. (2021) [41]	Depression	6.6	5.2	64	5.2	4.7	64	<0.05	Clinician guided: 74,4%
Perri et al. (2021) [42]	Depression	21.7	9.6	19	9.3	9.9	19	<0.0001	Clinician guided: 90.4%
	Anxiety	47.2	12.2	19	29.8	11.8	19	<0.0001	
Bantjes et al. (2021) [43]	Depression	14.0	3.6	125	8.5	5.0	125	<0.001	Clinician guided: 71.4%
	Anxiety	14.1	3.5	125	6.9	4.2	125	<0.001	
Liu et al. (2021) [34]	Depression	15.28	2.23	51	6.86	1.77	51	<0.001	N/I
	Anxiety	14.26	2.31	51	6.10	2.04	51	<0.001	
Al-Alawi et al. (2021) [35]	Depression	N/I	N/I	22	N/I	N/I	22	0.06	N/I
	Anxiety	N/I	N/I	22	N/I	N/I	22	0.01	

## Data Availability

Not applicable.

## Appendix A

Preferred Reporting Item for Systematic Review and Meta-analysis (PRISMA) Checklist.
